# How do cooperative rebates and asset specificity impact operating performance of farmer cooperatives? evidence from China

**DOI:** 10.1371/journal.pone.0313483

**Published:** 2024-11-08

**Authors:** Lijun Zeng, Junyi Wan

**Affiliations:** 1 School of Finance and Economics, Guangdong Polytechnic Normal University, Guangzhou, China; 2 College of Economics and Management, South China Agricultural University, Guangzhou, China; Universiti Tun Hussein Onn Malaysia, MALAYSIA

## Abstract

Improving farmer cooperatives’ operating performance is essential for their economic sustainability and for promoting sustainable agricultural development. Despite the prominent role of benefit distribution and asset specificity in cooperation organizations, their impact on performance remains controversial, and the relationship between the three is unclear. This study focuses on cooperative benefit distribution in terms of their rebates (first and second rebates) and investigates the relationship between asset specificity, cooperative rebate and operating performance. The results show that both the first and second rebates have a positive impact on operating performance and play substitutive roles in increasing operating performance. Physical asset specificity (PAS) impacts first rebates positively, whereas human asset specificity (HAS) impacts first rebates negatively. PAS has a positive effect on operating performance whereas HAS has no significant influence on operating performance. Furthermore, the first rebate mediates the relationship between PAS and operating performance and suppresses the relationship between HAS and operating performance. These findings provide support for developing relevant policies to improve cooperative performance and sustainability.

## 1.Introduction

As important organizations that link smallholder farmers to the market, farmer cooperatives (hereafter referred to as ‘cooperatives’) play increasingly important roles in sustainable agricultural development, as evidenced by their contributions to advancing the adoption of new technology and environmentally friendly practices [1,2], improving food quality and safety [3], and enhancing farmers’ welfare [4]. However, cooperatives face numerous internal and external challenges that impact their sustainability and roles in sustainable agricultural development [5], such as increased heterogeneity in member attitudes and objectives related to organizational growth [6,7], tight profit margins, competition, high volatility in commodity price [8]. How to effectively improve their management quality and operating performance is a major issue currently confronting cooperative development.

Benefit distribution, according to incentive theory, is a key management tool for shaping member cooperation behavior, which is critical to cooperative performance because cooperative survival and success are dependent on member collective actions [9]. To ensure member cooperation incentives and benefits, the International Cooperative Alliance advocated patronage refunds, which distribute surplus to members based on their transactions. In China, this is also known as the second rebate (*Erci Fanli*), which corresponds to the first rebate *(Yici Fanli*, in which cooperatives offer profit concessions to members in transactions such as purchasing member products at a higher than market price) [10]. However, there is increasing evidence of uneven benefit distribution in cooperatives, especially in many developing countries where the vast majority of members are smallholder farmers who are cooperative users and beneficiaries but not controllers or financers [11,12]. Benefit distribution is more likely to be skewed towards core members (controllers or financiers) in the form of being distributed based on capital, threatening the cooperation longevity and the sustainable performance of cooperatives [9]. For example, China explicitly requires cooperatives to return more than 60% of profits to members in proportion to their transactions in the Farmer Cooperative Law. However, in fact, most cooperatives distribute a large portion of profits based on capital shares rather than transaction volumes [13], and first rebates are frequently used to replace second rebates. Thus, some academics have advocated for additional measures to increase the standardization of cooperatives’ second rebates in order to support their growth [10]. Others, however, claim that the existence of practice has rationale, arguing that too many second rebates may harm cooperative performance by discouraging core member investment, which can easily lead to a lack of capital or even difficulties in cooperative operations [14]. These discussions are primarily theoretical, and little empirical research has been conducted to investigate the relationship between first and second rebates and operating performance. The first and second rebates both serve as member cooperation incentives; do they promote cooperative performance? Is there a substitution or complementarity between them? These questions are important and urgent research gaps that need to be filled.

Asset specificity is a crucial factor in determining cooperation benefit distribution and performance, and it has been extensively studied. However, there are still certain gaps in understanding in the literature about the role of asset specificity. First, the majority of research focuses on the role of asset specificity in the context of firms and interfirm cooperation, and whether it plays positive or negative roles in organizational performance is still debatable [15]. Second, the existing literature disregards the distinction of different asset specificities [16], as well as the role of benefit distribution in shaping asset specificity’s effects on performance. Indeed, the linkage between asset specificity and performance is strongly dependent on the ‘situation’ in which such investments are made [17]. Cooperatives are distinct from interfirm cooperation. They are characterized as one-to-many collaboration relationships deeply embedded in the rural social network between single cooperative enterprises, as legal entities, and multiple farmer members, as natural persons. Due to the high asset specificity of agricultural production [18], cooperatives must make asset-specific investments to improve operation efficiency and performance. However, according to resource-based theory and transaction cost theory, these investments may bring not only high rewards but also the risk of opportunistic exploitation and cooperation [19,20]. This may motivate the cooperative to rebate more to foster member cooperation and safeguard asset-specific investments. In the context of cooperatives, how does asset specificity affect cooperative rebates and operating performance?

To address these research gaps and questions, two distinct dimensions of asset specificity are distinguished in this study: physical asset specificity (PAS) with “hard” asset characteristics and human asset specificity (HAS) with “soft” asset characteristics [15]. This paper aims to explore the relationship between asset specificity, cooperative rebates and operating performance using face-to-face survey data from China’s cooperatives. Specifically, we examine the separate and joint impact of first and second rebates on cooperative operating performance, and further explore how PAS and HAS differentially impact cooperative rebates and operating performance.

This study contributes to the literature in three ways. First, this study goes beyond existing research on cooperative benefit distribution by arguing that both first and second rebates can improve operating performance and operate as substitutes. Previous research has primarily concentrated on the theoretical discussion over whether second rebates in cooperatives can improve their performance, with few studies exploring the critical role of first rebates in cooperatives. We empirically examine the separate and joint roles of first and second rebates in operating performance, and thus providing a more detailed and comprehensive understanding of the role of cooperative benefit distribution. Second, despite the fruitful discussion of the linkage between asset specificity and performance, there is a lack of understanding of how asset specificity affects operating performance in cooperative contexts. Based on the unique benefit distribution system of cooperatives, we introduce cooperative rebates as a critical mediating mechanism to reveal how asset specificity affects operating performance. In doing so, we offer new insights into the “black box” of how asset specificity impacts performance in cooperatives. Third, by examining the different effects of PAS and HAS on cooperative rebates and performance, we reveal that PAS has a positive impact on both first rebate and operating performance, while HAS has no effect on operating performance and a negative influence on first rebate. Therefore, this study not only responds to calls for more research on the differences between different types of asset specificity and offers a compelling explanation for the inconsistent findings on the relationship between asset specificity and performance and, but also contributes to the literature on the antecedents of cooperative rebates.

## 2. Theoretical analysis and research hypothesis

### 2.1 Cooperative rebates and operating performance

Cooperatives are collective action organizations in which members join to benefit from organizational advantages such as economies of scale to achieve cooperative surplus and thus maximize Pareto improvement. Their cooperative surplus primarily results from the price premium of joint marketing of agricultural products and the cost savings of joint procurement of agricultural inputs [21]. This is heavily reliant on how well their members cooperate. Cooperatives are more likely to achieve higher profits if members are dedicated to their growth and purchase agricultural inputs and supply agricultural products from them as cooperative needed. However, cooperatives are susceptible to opportunism and conflict problems due to their collective nature [22,23], such like side-selling to higher bidding buyers [24,25], investing less efforts in quality provision [26], which will negatively affect cooperative performance [27].

The human resource incentive theory proposes that an organization can inspire their member motivation by providing reasonable incentives. Cooperatives could offer benefit incentives to effectively promote member cooperation [28,29]. Since members remain independent entrepreneurs seeking individual economic benefits from cooperatives, whether they adopt some cooperation behaviors is primarily determined by the cost-benefit trade-offs [30]. Benefit incentives can promote member cooperation by raising the benefits of cooperation behavior and the opportunity costs of default. The first and second rebates, which can provide members with direct economic benefits, are the most significant and effective forms of benefit incentives for cooperative to members. If members do not receive reasonable rebates, they may turn to opportunistic behavior to boost their individual earnings or even quit the cooperative, which harms cooperative performance.

The first rebate has a “immediate incentive” feature, which can cause members to earn more than non-cooperative farmers by allowing members to obtain cheaper agricultural inputs or sell product at a better price during the transaction process. This contributes to cooperatives becoming more appealing to farmers, increasing member size and the scale of cooperative business, and thus improving cooperative scale advantage [10]. Simultaneously, this can ensure the quality of agricultural inputs for members [31], stabilize the yield and quality of members’ agricultural products, and thus improve the cooperative’s operating performance.

In comparison to the first rebate, the second rebate has the “anticipatory incentive” feature. The member can earn additional economic income by distributing the cooperative’s year-end surplus based on their transaction volume with the cooperative. This is something that an investor-owned firm cannot deliver (because dividends are distributed to external shareholders) [32]. A second rebate may result in a closer “bundle of benefits” relationship between cooperatives and their members. This contributes to member satisfaction and commitment [33], hence fostering cooperation behavior and cooperative operating performance [34]. The first and second rebates may therefore have a more positive impact on operating results. Thus, we hypothesize the following:

H1: First rebates (H1a) and second rebates (H2b) are positively related to operating performance.

First and second rebates may have complementary relationships in improving cooperative operating performance because of their complementary advantages in motivating members. On the one hand, the first rebate has the advantage of being simple to implement and providing a quick incentive for farmers to join and members to trade with cooperatives [10]. This can compensate for the shortcomings of second rebates in terms of their cumbersome operation and relatively slow incentive effect. The second rebate, on the other hand, has obvious advantages in encouraging members to pay attention to the cooperative’s long-term operation [33]. This can compensate for the first rebate’s shortcomings in motivating members to care about the cooperative’s future developments.

We contend that first and second rebates in China’s cooperatives are more likely to exhibit a substitution relationship in improving operating performance for two reasons. To begin, the first rebate is essentially an early concession of the cooperative operating profit. Under the limited rebate conditions of given cooperation cycles [13], cooperatives usually prioritize the implementation of first rebates with immediate incentives based on the industry price level to ensure business scale. This makes second rebate space for cooperatives mostly insufficient or even absent, making it difficult to fully utilize the incentive advantages of first and second rebates [35]. Second, even if some cooperatives perform well and have adequate rebate conditions during the cooperation cycle, they tend to provide insufficient second rebates due to the “rigid” nature of rebates that are easy to increase but difficult to decrease. As a result, an increase in one rebate boosts member collaboration incentive while lowering the marginal promoting effect of the other rebate on their cooperation behavior, hence weakening the promotion effect of the other rebate on cooperative operating performance. Accordingly, this study hypothesized the following:

H2: First and second rebates have substitution effects in terms of operating performance; that is, enhancing either rebate weakens the positive impact of the other on operating performance.

### 2.2 Asset specificity and operating performance

Asset specificity is defined as ‘the degree to which an asset can be redeployed to alternative uses by alternative users without sacrifice of productive value’ [36]. Asset specificity can be either tangible (such as physical materials such as tools and machinery) or intangible (such as knowledge-specific assets such as personnel training and development) [37]. However, most studies treat asset specificity as a generic construct, neglecting potential differences in the impact mechanism and relative importance of each dimension, which may thus result in suboptimal investments in each type of asset specificity [38]. Cooperatives’ main tasks in cooperative-member cooperation are to provide members with various services throughout the agricultural industry chain, such as technology training, market information, joint procurement of agricultural inputs, joint processing and marketing of agricultural products. To increase market competitiveness and cooperation surplus, they invest in a series of specific tangible and intangible assets, such as agricultural machinery, production technology and processing equipment. We focus our examinations on the PAS and HAS of cooperatives, drawing on the research of Wan et al. [16].

Asset specificity has both a positive value creation role and a negative opportunistic value expropriation role. First, according to the resource-based theory, specific assets are organizational “nuclear resources” with the characteristics of being valuable, rare, inimitable, and non-substitutable, which can improve organizational core competitiveness [39], resulting in higher performance. Second, according to relational exchange theory, asset specificity improves performance because it demonstrates the investor’s intention and commitment to engage in the transaction with a long-term orientation, which may encourage cooperation behavior [40]. Third, transaction cost theory emphasizes the negative role of specific assets, claiming that higher asset-specific investments put investors at lock-in risk and raise the possibility of opportunism and expropriation by the other party [41]. This is because the value of asset-specific investments is dependent on the transaction’s continuity with a specific partner, making them less valuable in alternative uses [36]. Thus, the relationship between asset specificity and organizational performance is ultimately determined by comparing the positive and negative effects of asset specificity.

We argue that both PAS and HAS may improve cooperative operating performance in China. First, the space for members to act opportunistically is arrowed by relatively lower information asymmetry between cooperatives and members. This is because China’s cooperatives are small and localized, and their managers and members are usually well acquainted with one another [42]. Second, cooperatives in China are embedded in rural acquaintances’ social networks, where social norms can effectively restrain farmers’ opportunistic behavior by making it more costly due to the threat of social sanctions and reputational consequences [43,44]. Cooperatives can effectively mitigate the negative effects of asset specificity given their current low asset specificity. The positive effects of cooperative asset specificity are likely to outweigh the negative effects. Thus, we propose the following:

H3: PAS (H3a) and HAS (H3b) are positively associated with cooperative operating performance.

### 2.3 Asset specificity and cooperative rebates

The role of various types of asset specificity in value creation and opportunistic value expropriation differs. These differences may result in varying demands for rebates with the function of motivating member cooperation. PAS has a higher propensity to induce partners’ opportunistic behavior and result in opportunistic value expropriation. This is because physical specific assets are tangible assets with “hard” and high “locking” characteristics, which are difficult and costly to transfer to other uses [45]. The larger the cooperative PAS, the greater the risk of opportunism and the potential loss [46,47]. This will motivate cooperatives to rebate more to foster member cooperation behaviors and increase member size, safeguarding against such risk and accelerating the cost-recovery of such investment. Additionally, cooperative PAS increases its reliance on members [48] and places it in a weak bargaining position with respect to benefit distribution [28,49]. Members are thus more likely to actively bargain for increased rebates. Thus, we propose the following:

H4: PAS is positively associated with the first rebates (H4a) and second rebates (H4b).

We contend that HAS may reduce the need for rebates for the following reasons. First, HAS increases partner cooperation dependence because human-specific assets are intangible assets with “soft” and high “unique competitiveness” characteristics that are more valuable, scarce, and difficult to replace [50]. When cooperatives invest more in human-specific assets, they can offer more appealing “soft” services to farmers with limited management capability and education, such as technical training, information consultation, and management guidance. Under such conditions, the need and reliance of members and farmers to cooperate with the cooperative increases, which inhibits their opportunism and thus lessens the need for rebate incentives. Second, human asset-specific investments are typically long-term oriented, signaling investors’ long-term collaboration commitment [51]. It can increase partner trust and encourage interaction and information sharing, allowing investors to better understand and solve specific problems [16]. Furthermore, greater HAS increases the likelihood of establishing a good cooperative environment by providing more frequent and better “soft” services. As a result, when the specificity of cooperative human assets is high, their members are more likely to act collaboratively rather than opportunistically to exploit cooperative lock-in situations, thereby reducing the possibility for cooperatives to use rebate incentives. Thus, we propose the following:

H5: HAS is negatively associated with first rebates (H5a) and second rebates (H5b).

### 2.4 Mediating and suppressing effect of cooperative rebates

Resources alone are insufficient for cooperatives [52]. To maximize resource value and enhance organizational performance, cooperatives must mobilize all members to collaborate to use resources effectively and prevent the negative impacts of opportunism caused by asset specificity. Cooperative rebates, as the key to influencing members’ cooperation behavior, serve as a critical “bridge” between asset specificity and organizational performance. However, cooperative rebate mediating mechanisms differ due to differences in PAS and HAS.

As discussed above, the resource flexibility of physical specific assets is poor, with a high “locking” effect. This increases the risk of cooperatives being exposed to opportunistic behaviors and cooperation risks [16], necessitating higher cooperative rebates to encourage member cooperation and safeguard physical specific assets to improve organizational performance. As a result, PAS may improve operating performance by increasing cooperative rebates.

Because of the high “unique competitiveness” and “long-term oriented” characteristics of human-specific assets [51], cooperatives’ HAS can not only improve member dependence on cooperatives’ services or business but also promote member trust in cooperatives and interaction between the cooperatives and members. These can encourage member cooperation and thus reduce the need for cooperative rebate incentives. As a result, the true positive effect of HAS on operating performance is likely to be suppressed by the indirect effect of cooperative rebates. In other words, cooperative rebates may suppress, rather than mediate, the influence of HAS on operating performance. Given the analysis above, the following was hypothesized:

H6. First rebates (H6a) and second rebates (H6b) mediate the relationship between PAS and operating performance.
H7. First rebates (H7a) and second rebates (H7b) suppress the relationship between HAS and operating performance.

**Fig 1:**
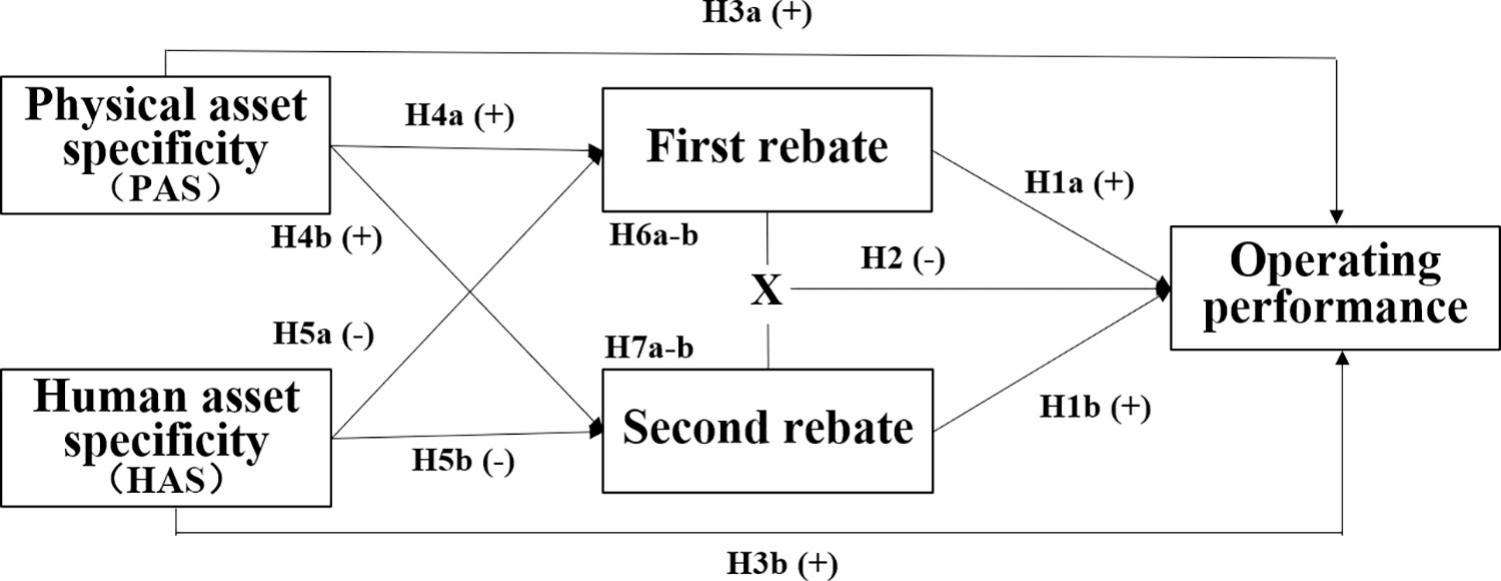
The proposed research model.

The proposed research model is shown in Fig 1.

## 3. Materials and methods

### 3.1 Data sources and sample characteristics

The data were gathered through a face-to-face survey of cooperatives in Guangdong, Jiangxi, Guizhou and Guangxi provinces in China. To improve the sample’s representativeness and validity, we randomly selected seven prefectural-level cities from the four provinces in China’s eastern, central, and western regions as the survey area, taking into account regional differences and field survey feasibility. Then, we randomly chose sample cooperatives from a list of cooperatives collected from government websites in these survey areas. Several steps were taken to further ensure the accuracy of survey data. First, we conducted two pre-surveys in Guangdong Province to finalize the final questionnaire after several rounds of revisions. Second, a structured questionnaire was used to interview the chairperson and was completed by trained investigators based on the chairperson’s responses. The data collection process fulfilled all ethical norms and standards of the research. Verbally informed consent was obtained from all respondents involved in our study. This survey distributed 309 questionnaires, and 227 valid samples were obtained after eliminating false cooperatives and questionnaires with missing key information, yielding an effective rate of 73.46%.

Table 1 shows the sample characteristics. The sample cooperatives have an average age of 8.23 years, with the oldest being 14 years. This is consistent with the fact that the number of cooperatives in China has been rapidly increasing since 2007, as well as roughly with the sample cooperatives of Liang et al. [34], which have a maximum establishment time of 13 years. The average shareholding ratio of chairpersons reached 50.99%. This is in line with the findings of studies such as Liang and Hendrikse [53] and Huang and Liang [13], which revealed that chairperson or a few core members control the majority of cooperative shareholding in China’s cooperatives.

**Table 1:** Sample characteristics.

Item	Category	Frequency	Proportion / %	Item	Category	Frequency	Proportion / %
Age of cooperatives	≦2	9	3.96	Chairperson’s capital shares	≦20%	66	29.07
	2~5	38	16.74		20%~50%	55	24.23
	5~8	65	28.63		50%~80%	55	24.23
	8~11	89	39.21		>80%	51	22.47
	>11	26	11.45	Alliance types	“Cooperative+Farmers”	92	40.53
					“Company+Cooperative+Farmers”	135	59.47
Membership size	≦50	44	19.38	Demonstration level of cooperatives	Non-demonstration	25	11.01
	50~100	56	24.67		County-level	12	5.29
	100~150	70	30.84		Municipal-level	44	19.38
	150~200	25	11.01		Provincial-level	94	41.41
	>200	32	14.1		National-level	52	22.91

**Note:** A demonstration cooperative is a type of cooperative that is selected and grants by the government based on its performance in terms of development and operation norms, economic strength, and social reputation [29]. It consists of four levels: national, provincial, municipal, and county, with the selection criteria decreasing in turn and having to follow the application order from county to national level step by step.

### 3.2 Variable definition

Objective and subjective approaches for measuring cooperative performance are available [54]. Subjective methods are more suited to evaluating China’s cooperative performance [55]. On the one hand, the financial statements of China’s cooperatives are often unsound and irregular, making it difficult to obtain complete and valid data on financial indicators of performance; on the other hand, the operating profits of cooperatives measured in monetary terms may not be comparable due to differences in regional development and product characteristics [55,56]. Therefore, we draw on the research of Wan et al. [56] to use the sum of the subjective evaluation scores of the two items to measure cooperative operating performance: operating profit and loss, and profitability. Operating profit and loss can visually reflect organizational operating performance and is an important indicator of short-term performance. Profitability reflects the organization’s ability to earn profits and is a key indicator of organizational performance [57], especially long-term performance. Referring to Artz and Brush [58] and Yu [59], PAS and HAS were measured by the level of losses suffered by cooperatives from investment in specialized equipment, knowledge and skills when they no longer operate the existing business, respectively. Following Ren and Yu [10], the first rebate was measured by “whether cooperatives offer joint purchasing or marketing services, allowing farmer-members to purchase input at a cheaper price or sell their products at a higher price than the market price”. “Weather cooperatives allocate profit to members based on transaction volumes” was used to measure the second rebate based on Han et al. [35]. Organizational characteristics are important in determining organizational behavior and the outcomes of that behavior. With reference to previous research [34,56], we used organizational characteristics factors as control variables, including cooperative age, membership size, chairperson’s capital shares, alliance types, and demonstration level of cooperative. Table 2 shows the specific definitions and statistical descriptions of these variables.

**Table 2:** Definition of variables and summary statistics.

Variables	Definition	Mean (SD)
Operating performance	Total scores of the two items: operating profit and loss over last two years (5 = large profit, 4 = more profit, 3 = basically no profit, 2 = more loss, 1 = large loss or no for-profit operations); profitability of cooperatives in comparison to their peers (5 = much better, 4 = better, 3 = almost equal, 2 = a little bit worse, 1 = much worse or no for-profit operations)	7.37 (1.97)
PAS	The degree of losses suffered by cooperatives in terms of equipment and machine tool investments if they no longer operate their existing business (5 = very high, 4 = relatively high, 3 = general, 2 = relatively low, 1 = very low)	3.36 (1.34)
HAS	The degree of losses suffered by cooperatives in terms of investment in knowledge and skills if they no longer operate their existing business (5 = very high, 4 = relatively high, 3 = general, 2 = relatively low, 1 = very low)	2.85 (1.25)
First rebate	1 = if cooperatives offer joint purchasing or marketing services, allowing farmer-members to obtain input at a lower price or deliver their products at a higher price than the market price, 0 = otherwise	0.82 (0.39)
Second rebate	1 = if cooperatives distribute profit to members based on transaction volumes, 0 = otherwise	0.33 (0.47)
Age of cooperatives	Logarithm of cooperatives’ age in 2018	2.01 (0.50)
Membership size	Logarithm of the number of members in a cooperative	4.54 (0.96)
Chairperson’s capital shares	The shareholding ratio of cooperative chairperson in 2018	50.99 (33.00)
Alliance types	1 = “Company+Cooperative+Farmers”, 0 = “Cooperative+Farmers”	0.59 (0.49)
Demonstration level of cooperatives	5 = national-level, 4 = provincial-level, 3 = municipal-level, 2 = county-level, 1 = non-demonstration	3.60 (1.21)

## 3.3 Methodology

To examine the influence of first and second rebates on operating performance, we developed the following empirical model.


$$Perf=c_{1}+a_{1}Freb+a_{2}Sreb+a_{3}X_{j}+\varepsilon_{1}$$ (1)

In Eq (1), *Perf* refers to operating performance, *Freb* and *Sreb* represent the first and second rebates, respectively, and *X*_*j*_ is a series of control variables.

Following Mishra and Nielsen [60], this study used interactive regression coefficients to test the substitution effect of first and second rebates, and the following econometric model was developed:

$$Perf=c_{2}+a_{4}Freb+a_{5}Sreb+a_{6}(Freb\times Sreb)+a_{7}X_{j}+\varepsilon_{2}$$ (2)

In Eq (2), if *α*_6_ is significantly negative, it indicates that the marginal effect of the first or second rebates decreases as the other party increases, and the two play a substitutive role in operating performance.

Referring to Baron and Kenny [61] and MacKinnon et al. [62], the causal steps approach was used to further analyze the relationship between asset specificity, rebates, and operating performance to test H3 ~ H7. The empirical models are expressed as follows:

$$Perf=c_{3}+\beta_{1}Masp+\beta_{2}Hasp+\beta_{3}X_{j}+\varepsilon_{3}$$ (3)

$$Freb=c_{4}+\beta_{4}Masp+\beta_{5}Hasp+\beta_{6}X_{j}+\varepsilon_{4}$$ (4)

$$Sreb=c_{5}+\beta_{7}Masp+\beta_{8}Hasp+\beta_{9}X_{j}+\varepsilon_{5}$$ (5)

$$Perf=c_{6}+\beta_{10}Freb+\beta_{11}Masp+\beta_{12}Hasp+\beta_{13}X_{j}+\varepsilon_{6}$$ (6)

$$Perf=c_{7}+\beta_{14}Sreb+\beta_{15}Masp+\beta_{16}Hasp+\beta_{17}X_{j}+\varepsilon_{7}$$ (7)

In Eqs (3) to (7), *Masp* and *Hasp* refer to PAS and HAS, respectively. Eq (3) was conducted to examine the impact of asset specificity on operating performance. Eqs (4) and (5) were used to investigate the influence of asset specificity on the first and second rebates, respectively. The indirect effect of the first rebate between asset specificity (PAS and HAS) and operating performance was investigated using Eqs (3), (4) and (6). Eq (3) investigates the overall effect of the independent variable (PAS/ HAS) on the dependent variable (operating performance). Eq (4) evaluates the effect of the independent variable on the indirect variable (first rebate). Eq (6) investigates the effects of both the independent and indirect variables on the dependent variable. If both *β*_4_(*β*_5_) and *β*_10_ are significant, there is a significant indirect effect between PAS (HAS) and operating performance. If only one of *β*_4_(*β*_5_) or *β*_10_ is significant, the Bootstrap method should be used to test the significance of the indirect effect between PAS (HAS) and operating performance. If both the indirect effect (*β*_4_*β*_10_) and the direct effect (*β*_11_) are significant and the sign of two are consistent, first rebate has a mediating effect between PAS and operating performance. If both the indirect effect (*β*_5_*β*_10_) and the direct effect (*β*_12_) are significant and the sign of two are opposed, first rebate has a suppressing effect between HAS and operating performance. Furthermore, first rebate suppresses the relationship between HAS and operating performance, provided that the indirect effect (*β*_5_*β*_10_) is significant and the direct effect (*β*_12_) and the total effect (*β*_2_), are not significant. The indirect effect of the second rebate between asset specificity and operating performance was investigated using Eqs (3), (5) and (7). The analysis procedure is the same as the first rebate’s indirect effect test. The Order Probit model was utilized to estimate Eqs (1) to (3) and (6) to (7), while the Probit model was used to estimate Eqs (4) and (5) because operating performance is an ordered variable, and first and second rebates are binary variables.

## 4. Results and discussion

### 4.1 Effect of cooperative rebates on operating performance

To ensure the validity and consistency of the model estimations, we first employed the variance inflation factor (VIF) to determine whether the variables were multicollinear. The results reveal that the VIF values for all variables are less than 1.8, indicating that multicollinearity is not an issue in this study. The models were then estimated with heteroskedasticity robust standard errors to eliminate the possibility of heteroskedasticity interference.

Table 3 displays the effects of cooperative rebates on operating performance. As Model 1 in Table 3 shows, the regression coefficients of first and second rebate are significantly positive (beta = 0.423, p < 0.05; beta = 0.343, p < 0.05), indicating that both the first and second rebates have a positive impact on operating performance. H1a and H1b are therefore supported. The result of the second rebates is in line with the findings of previous studies, which found that profit distribution based on patronage has positive effects on cooperative economic performance [34]. The average marginal effects of the first and second rebates were calculated further to compare their impact on operating performance. According to the results in Table 4, cooperative first rebates can reduce the probability of their operating performance being at levels 2, 5, 6, and 7 by 4.70%, 3.50%, 3.70%, and 3.00%, while increasing the probability of their operating performance being at levels 8, 9 and 10, respectively, by 2.00%, 5.80% and 7.70%. Furthermore, cooperative second rebates can reduce the probability of their operating performance being at levels 2, 5, 6, and 7 by 3.80%, 2.90%, 3.00%, and 2.40%, while increasing the probability of their operating performance being at levels 8, 9 and 10, respectively, by 1.60%, 4.70% and 6.30%. These results imply that cooperative first or second rebate can decrease the likelihood of lower operating performance (being at the levels of 2 to 7) while increasing the probability of greater performance (being at the levels of 8 to 10). Additionally, at all levels of operating performance, the average marginal effect of the first rebate has a higher absolute value than the second rebate. This implies that the first rebate has a greater positive impact on operating performance than the second rebate, and members are more motivated to commit to cooperative development by the first rebate. Farmers are typically risk averse and are more concerned with short-term cash returns. The first rebate enables members to quickly acquire consistent rebate income and avoid some management risk, whereas the second rebate is influenced by market conditions and has a very high level of uncertainty. First rebates are therefore more appealing and give members a stronger incentive to cooperate than second rebates. According to the results of Model 2 in Table 3, the regression coefficient of interaction terms for the first and second rebates is significantly negative (beta = –1.264, p < 0.01). This reveals that the positive impacts of the first and second rebates on operating performance can be substituted for one another. In other words, increasing one of the two rebates reduces the positive effect of the other on operating performance. H2 is therefore supported. Due to the inadequate rebate capability of the majority of China’s cooperatives and the “rigidity” of rebates, it is difficult for the first and second rebates to play a complementing incentive function in farmer cooperation behavior. Because of this, there is a link between the two types of rebates’ influence on operating performance: “as one grows, another drops”.

**Table 3:** Estimation results of Eqs (1) and (2).

Variable	Model 1		Model 2	
	Coeff.	Robust Std. Err.	Coeff.	Robust Std. Err.
First rebate	0.423**	0.201	0.275	0.216
Second rebate	0.343**	0.152	0.425***	0.161
First rebate×second rebate	—	—	-1.264**	0.539
Age of cooperatives	-0.515***	0.161	-0.540***	0.161
Membership size	0.093	0.080	0.087	0.081
Chairperson’s capital shares	0.004*	0.002	0.003	0.002
Alliance types	0.153	0.148	0.115	0.150
Demonstration level of cooperatives	0.184**	0.074	0.181**	0.074
Pseudo R^2^	0.039		0.048	
Wald chi2	38.090***		41.070***	

Note

*, P ≤ 0.10

**, P ≤ 0.05

***, P ≤ 0.01.

**Table 4:** Average marginal effects of the oprobit model 1.

Operating performance	Perf = 2	Perf = 4	Perf = 5	Perf = 6	Perf = 7	Perf = 8	Perf = 9	Perf = 10
First rebates	-0.047*	-0.005	-0.035**	-0.037**	-0.030**	0.020*	0.058**	0.077**
	(0.025)	(0.004)	(0.018)	(0.019)	(0.015)	(0.012)	(0.028)	(0.038)
Second rebates	-0.038**	-0.004	-0.029**	-0.030**	-0.024**	0.016*	0.047**	0.063**
	(0.019)	(0.004)	(0.014)	(0.014)	(0.011)	(0.009)	(0.021)	(0.029)

**Note:** Robust standard errors in parentheses

*, P ≤ 0.10

**, P ≤ 0.05

***, P ≤ 0.01.

### 4.2 Effect of asset specificity on cooperative rebates

Table 5 represents the estimation results for Models 3 through 7. The regression coefficients of PAS and HAS in Model 4 are significantly positive and negative (beta = 0.239, p < 0.05; beta = –0.185, p < 0.10), respectively. This reveals that PAS has significant positive effects on the cooperative’s first rebate, whereas HAS has significant negative effects. As a result, H4a and H5a are both supported. These results support previous research showing that it is critical to differentiate between the asset specificity of various types and that, as a result of their variations, PAS and HAS may have different effects on organizations [38]. The estimation results of Model 5 in Table 5 show that the effects of PAS and HAS on second rebates are insignificant (beta = 0.032, p > 0.10; beta = –0.034, p > 0.10), so neither H4b nor H5b is supported. The reason for the insignificant effect of asset specificity on second rebates may be that China’s cooperatives favor first rebates over second rebates as an incentive for farmers to actively cooperate. Given the limited space for rebates in an increasingly competitive market, cooperatives prefer to use first rebates to quickly improve their attractiveness to motivate farmers to cooperate, thereby increasing their business scale and operating performance. As the PAS rises, cooperatives may seldom boost second rebates to safeguard it by encouraging member cooperation. When HAS increases, cooperatives become more appealing to members, but this may not reduce the requirement for a second rebate with a cooperative incentive function. As a result, second rebates are unlikely to rise with PAS, and there is little probability that second rebates will reduce as HAS increases.

**Table 5:** Estimation results of Eqs (3)–(7).

Variable	Operating performance			First rebate		Second rebate	
	Model 3	Model 6	Model 7		Model 4		Model 5
PAS	0.151**	0.130**	0.149**		0.239**		0.032
	(0.066)	(0.066)	(0.066)		(0.097)		(0.081)
HAS	-0.025	-0.009	-0.021		-0.185*		-0.034
	(0.070)	(0.069)	(0.070)		(0.109)		(0.088)
First rebate	—	0.411**	—		—		—
		(0.203)					
Second rebate	—	—	0.373**		—		—
			(0.149)				
Age of cooperatives	-0.503***	-0.492***	-0.497***		-0.211		-0.119
	(0.176)	(0.170)	(0.174)		(0.258)		(0.231)
Membership size	0.098	0.088	0.097		0.102		-0.014
	(0.080)	(0.079)	(0.080)		(0.108)		(0.095)
Chairperson’s capital shares	0.003	0.003	0.003		-0.006**		-0.005*
	(0.002)	(0.002)	(0.002)		(0.003)		(0.003)
Alliance types	0.188	0.175	0.157		0.169		0.279
	(0.148)	(0.148)	(0.148)		(0.205)		(0.185)
Demonstration level of cooperatives	0.210***	0.198***	0.180**		0.150		0.282***
	(0.075)	(0.073)	(0.077)		(0.099)		(0.096)
Pseudo R^2^	0.034	0.0399	0.0412		0.069		0.060
Wald chi2	25.520***	32.470***	34.620***		15.520**		17.290**

**Note:** Robust standard errors in parentheses

*, P ≤ 0.10

**, P ≤ 0.05

***, P ≤ 0.01.

### 4.3 Effect of asset specificity on operating performance

#### 4.3.1 Main effect test

The results of Model 3 in Table 5 show that the coefficient of PAS is significantly positive while the coefficient of HAS is not (beta = 0.151, p < 0.05; beta = 0.025, p > 0.10). This suggests that while PAS can significantly improve cooperative operating performance, HAS has no significant relationship with operating performance. H3a is thus verified, but H3b is not supported. H3b did not pass the test, probably because of the existence of a suppressing effect. The suppressing effect of the third variable may result in an insignificant relationship between the independent and dependent variables [62]. The insignificant impact of HAS on operating performance could be attributed to being suppressed by the cooperative rebate, which we will analyze more in Hypothesis 7. Overall, asset specificity and operating performance are positively related. This somewhat validates Delbufalo’s claim that asset specificity improves performance in buyer-supplier relationships, which was confirmed by a meta-analysis [45].

#### 4.3.2 Mediating and suppressing effect test

First rebate was introduced into Model 6 to evaluate its mediating between PAS and operating performance and its suppressing effects between HAS and operating performance. The results in Table 5 show that both the coefficients of the first rebate and PAS are significantly positive (beta = 0.411, p < 0.05; beta = 0.130, p < 0.05). Additionally, the coefficient of PAS in Model 3 is significant and greater than that in Model 6, suggesting that the positive effect of PAS on operating performance is reduced after introducing the mediating variable of the first rebate. The above results implies that first rebates play a partially mediating role in the impact of PAS on operating performance. As a result, H6a is supported. The coefficient of first rebate in Model 6 and the coefficient of HAS in Model 4 is significant, suggesting that the indirect effect of first rebate on the relationship between HAS and operating performance is significant. Moreover, the coefficient of HAS in both Model 3 (beta = –0.025, p > 0.10) and Model 6 (beta = –0.009, p > 0.10) is not significant. These results indicates that first rebate play a suppressing effect on the relationship between HAS and operating performance. Thus, H7a is supported. The second rebate was added to Model 7 to assess its mediating and suppressing effects. The results of Model 7 in Table 5 show that the regression coefficients of second rebate and PAS are significant (beta = 0.373, p < 0.05; beta = 0.149, p < 0.05), but not the HAS’s coefficient (beta = –0.021, p > 0.10). Given that the impact of PAS on second rebates is not significant in Model 5 (beta = 0.032, p > 0.10), the Bootstrap method was conducted to further test the significance of the indirect effect of second rebates on the relationship between PAS and operating performance. The results show that the indirect effect of second rebate is not significant with a 95% confidence interval from –0.030 to 0.037 that did contain zero. This indicates that second rebate’s mediating role between PAS and operating performance is insignificant and H6b is thus not verified. Since the coefficient of HAS in Model 5 is not significant (beta = –0.034, p > 0.10) and the coefficient of second rebate in Model 7 is significant (beta = 0.373, p < 0.05), the Bootstrap method was used to investigate the indirect effect of second rebates on the linkage between HAS and operating performance. The results show that the 95% confidence interval for the indirect effect is -0.041 to 0.035, which includes zero. It can be concluded that second rebate has no significant suppressing role between HAS operating performance. Thus, H7b is not verified. To summarize, first rebates play a mediating role on the relationship between PAS and performance and a suppressing role on the relationship between HAS and performance. Second rebates are not significant in mediating the relationship between PAS and performance or in suppressing the relationship between HAS and performance. The insignificant indirect effect of second rebates could be attributed to the fact that China’s cooperatives are more likely to provide benefit incentives to members in the form of first rebates rather than second rebates. According to data from China’s Ministry of Agriculture and Rural Affairs, only 20.30 percent of the nation’s 116,000 cooperatives implemented second rebates in 2020. According to our survey data, the first and second rebates have mean values that are 0.82 and 0.33, respectively, above and below the median 0.5 of their respective value ranges. This implies that the majority of cooperatives in China offer their members first rebates, but fewer of them carry out second rebates, resulting in second rebates playing a minor indirect impact on the link between asset specificity and operating performance.

### 4.4 Endogeneity and robustness tests

The above empirical results indicate that specific investment and rebate behavior has a positive impact on cooperatives’ operating performance. However, due to data and variable restrictions, a sample selection bias may exist. For example, while cooperatives’ specific investment and rebate behavior influences operating performance, some cooperatives may engage in specific investment and rebate behavior when they feel their operating performance are well. To avoid potential sample selection bias and endogeneity issues interfering with the empirical results, we employed the propensity score matching (PSM) method to build a counterfactual framework of specific investment and rebate behavior on cooperatives’ operating performance, and then performed robustness tests and corrections to the regression results above. The results are shown in Table 6. The nearest-neighbor matching (k = 2 or 4) results showed that the PSM matching satisfied the balance test. The average treatment effects for PAS, first and second rebates are statistically significant at the 5% or 10% level and have positive coefficients. The average treatment effect of HAS, on the other hand, did not pass the significance test. This means that only HAS has no significant effect on cooperative operating performance, whereas PAS, first and second rebates all have a significant impact. This is consistent with previous empirical findings. We further used radius matching and kernel density matching methods to estimate their average treatment effects, and the results are still in line with the above. This demonstrates that our empirical results are robust.

**Table 6:** The results of propensity score-matching methods.

Matching method	PAS	HAS	First rebate	Second rebate
**Nearest-neighbor matching(n = 2)**	0.664**	0.407	0.903*	0.787**
**Nearest-neighbor matching(n = 4)**	0.537*	0.378	0.851*	0.750**
**Radius matching(r = 0.01)**	0.641**	0.294	1.025**	0.667**
**Kernel-based matching**	0.647**	0.421	0.930**	0.742***

Note

*, P ≤ 0.10

**, P ≤ 0.05

***, P ≤ 0.01.

To further ensure the reliability of the empirical results, we conducted robustness tests on the main effects in third aspects. First, we replaced the aforementioned Order Probit and Probit models with Order Logit and Logit models, respectively. The results in Table 7 show that the significance and coefficient signs of all core variables are consistent with previous empirical findings. Second, to avoid the influence of extreme values on model estimation, the Winsorize method is employed to assess the reliability of the results by smoothing the high and low 5% odd values of the key variables in the sample. The regression results shown in Table 8 are consistent with our main results in Tables 3 and 5, indicating that the empirical results in this study are robust.

**Table 7:** Estimation results of the Order Logit and Logit models.

Variable	Operating performance					First rebate	Second rebate
	Order Logit models					Logit models	
**First rebate**	0.693*	0.419	—	0.670*	—	—	—
	(0.385)	(0.426)		(0.371)			
**Second rebate**	0.488*	0.676**	—	—	0.553**	—	—
	(0.268)	(0.300)			(0.266)		
**First rebate×second rebate**	—	-2.204**	—	—	—	—	—
		(1.080)					
**PAS**	—	—	0.285**	0.255**	0.281**	0.409**	0.057
			(0.124)	(0.121)	(0.123)	(0.167)	(0.133)
**HAS**	—	—	-0.054	-0.033	-0.042	-0.323*	-0.051
			(0.127)	(0.126)	(0.129)	(0.192)	(0.145)
**Control variables**	Control	Control	Control	Control	Control	Control	Control
**Pseudo R** ^**2**^	0.0363	0.0444	0.0353	0.0402	0.0405	0.0668	0.0594
**Wald chi2**	32.600***	34.070***	27.910***	33.130***	33.070***	16.300**	16.170**

**Note:** Robust standard errors in parentheses

*, P ≤ 0.10

**, P ≤ 0.05

***, P ≤ 0.01.

**Table 8:** Estimation results of the Winsorize method.

Variable	Operating performance					First rebate	Second rebate
**First rebate**	0.427**	0.349*	—	0.414**	—	—	—
	(0.201)	(0.200)		(0.203)			
**Second rebate**	0.343**	0.408***	—	—	0.373**	—	—
	(0.152)	(0.154)			(0.149)		
**First rebate×second rebate**	—	-0.164**	—	—	—	—	—
		(0.067)					
**PAS**	—	—	0.152**	0.131**	0.149**	0.239**	0.032
			(0.066)	(0.066)	(0.066)	(0.097)	(0.081)
**HAS**	—	—	-0.026	-0.010	-0.022	-0.185*	-0.034
			(0.070)	(0.069)	(0.070)	(0.109)	(0.088)
**Control variables**	Control	Control	Control	Control	Control	Control	Control
**Pseudo R** ^**2**^	0.0389	0.0472	0.0338	0.0397	0.0409	0.0682	0.0598
**Wald chi2**	38.160***	43.430***	25.430***	32.310***	34.580***	15.420**	17.260**

**Note:** Robust standard errors in parentheses

*, P ≤ 0.10

**, P ≤ 0.05

***, P ≤ 0.01.

## 5. Conclusions and implications

Cooperatives stand out as promoting sustainable agricultural development, but their performance and sustainability face serious challenges. This study investigated how cooperative rebates and asset specificity affect operating performance. The findings revealed that a) both the first and second rebates have a significant positive impact on operating performance, with the first rebate having a stronger impact; b) the first and second rebates have a substitution relationship in promoting operating performance; c) PAS has a positive impact on the first rebate, while HAS has a negative impact on the first rebate, and both have no significant impact on the second rebate; d) PAS has a significant positive impact on operating performance, whereas HAS has no significant relationship with operating performance; and e) first rebate plays a mediating role in the relationship between PAS and operating performance and plays a suppressing role in the relationship between HAS and operating performance.

Our findings reveal some important implications for developing countries such as China to improve the operating performance and sustainable development of cooperatives. First, governments should reduce their intervention in the second rebates of cooperatives, and support and encourage cooperatives to flexibly enhance their first and second rebates to better promote the development of cooperatives. The second rebate is currently given far too much consideration in China’s Cooperative Law, while the potential replacement of the first and second rebates in the areas of interest distribution and incentive function is largely ignored. Our findings reveal that first and second rebates play substitutive roles in increasing operating performance, with the first rebate having a stronger positive impact on operating performance. Therefore, to fully capitalize on the promoting effects of the two rebate modes on operating performance, the government could slightly relax the legal proportion requirements of the second rebate, allow for flexible replacement of the rebate mode, and direct cooperatives to implement the first and second rebates in accordance with their development needs.

Second, cooperative managers should implement appropriate rebate behaviors based on the characteristics of asset-specific investments in order to maximize the promotional effect of asset specificity and rebate behaviors on operating performance. Our research provides evidence that different types of asset specificity and rebate behavior yield different outcomes in cooperatives. Specifically, our findings show that PAS has a positive impact on first rebates and operating performance, whereas HAS has a negative effect on first rebates but no significant effect on operating performance. Furthermore, first rebates outperform second rebates in terms of enhancing operating performance. As a result, we suggest that cooperatives not only improve PAS, first and second rebates to improve operating performance, but also focus on enhancing the matching of different forms of asset specificity and rebate behavior. When cooperatives make larger human asset-specific investments, the strength of the first rebate can be flexibly decreased to match their development. This is because they may provide more “soft” services with cooperation incentives for members, reducing their reliance on cooperation incentive functions of first rebates. Cooperatives with higher physical asset-specific investments should consider increasing the strength of the first rebate to encourage members’ cooperation and patronage. This will reduce PAS’s negative opportunistic value expropriation role, increase the cooperatives’ scale effect and PAS’s utilization rate, and ultimately improve operating performance.

Third, farmers should consider the asset specificity of cooperatives while selecting cooperative partners and negotiating benefit distribution. According to our research, cooperatives with higher PHA have greater first rebate and operating performance. Asset specificity plays a dual role in value creation and opportunistic value expropriation. Higher PHA not only enhances cooperative performance but also reinforces the cooperative’s reliance on its members and the necessity and degree of member incentives. Therefore, we suggest farmers to choose cooperatives with higher PHA for collaboration and negotiate to acquire higher cooperative rebates based on cooperative’s PHA in order to maximize their collaborative benefits. Additionally, our findings also reveal that HAS of cooperatives is negatively related to first rebate. This suggests that HAS with a “soft” competitive advantage serves the purpose of incentivizing member collaboration, reducing the need for cooperatives to utilize the first rebate to encourage member cooperation. As a result, we suggest that farmers consider the relative value of cooperative’s HAS and the first rebate to themselves depending on their personal situation while deciding which cooperative to join. In other words, while selecting a cooperative, members ought to weigh not just the amount of the first rebate but also the HAS’s contributions to their benefit.

Although our study yielded meaningful findings, some limitations and future directions warrant further discussions. First, this study examines the effect of asset specificity, rebate behavior and some organizational characteristics on cooperative performance only at the organizational level; nevertheless, in reality there are many other factors that may influence organizational performance. Examples include the organization’s external political and economic environment [63], as well as the governance mechanisms [64]. Future research could expand the scope of antecedent variables to further examine the impact of factors such as external environment and leadership style on cooperative operating performance. Second, our empirical data are cross-sectional and come exclusively from Chinese cooperatives, which may limit the generalizability of our research findings. Future research can collect longitudinal data as well as data from other nations or industries for dynamic comparison and analysis in order to expand on the findings of this study. Third, while this study focuses on the link and impact effect between asset specificity, cooperative rebates, and operating performance, it pays little attention to the boundaries of that relationship and effect. Future research may include integrating situational variables such as external environment, organizational, or membership characteristics to broaden the scope. For example, investigate how different cooperative life cycle stages or social embeddings influence the link between asset specificity, rebates, and operating performance.

## Supporting information

S1 File Research data. (XLSX)
